# The mediating role of self-care capacity in the relationship between perceived burden and quality of life among patients following mechanical valve replacement: a structural equation modeling analysis

**DOI:** 10.3389/fcvm.2026.1887530

**Published:** 2026-08-26

**Authors:** Yuanyuan Yang, Xiaotian Zhang, Yunqing Chen, Yina Zhao, Shuizhen Ou, Wenhui Li, Yingshan Zhou

**Affiliations:** 1 Department of Cardiac Surgery, Institute of Cardiovascular, Guangdong Provincial People's Hospital (Guangdong Academy of Medical Sciences), Southern Medical University, Guangzhou, China; 2 Cardiac Surgery Intensive Care Unit, The First Affiliated Hospital of Guangzhou Medical University, Guangzhou, China

**Keywords:** mechanical valve replacement, quality of life, self-care capacity, self-perceived burden, structural equation modeling

## Abstract

**Background:**

Patients undergoing mechanical valve replacement face lifelong physical and psychological challenges that affect quality of life (QoL). While self-perceived burden and self-care capacity are both associated with QoL, the mediating mechanism by which self-perceived burden influences QoL through self-care capacity remains incompletely understood—particularly among patients with mechanical valve replacement. Clarifying this pathway could inform targeted nursing interventions for this population.

**Aim:**

To examine the mediating role of self-care capacity in the relationship between self-perceived burden and QoL among patients with mechanical valve replacement, using structural equation modeling to evaluate both direct and indirect effects.

**Methods:**

A cross-sectional design was used with 464 patients from a tertiary hospital in China. Participants completed validated questionnaires: the Self-Perceived Burden Scale(SPBS), the Exercise of Self-Care Agency Scale(ESCAS), and the 36-Item Short Form Survey(SF−36). Self-care capacity was modeled as a mediator within the structural equation modeling framework. Bootstrapping with 5,000 resamples was used to verify the significance of the indirect effect.

**Results:**

Self-perceived burden was negatively correlated with both self-care capacity(*r* = −0.760, *P* < 0.001) and QoL(*r* = −0.826, *P* < 0.001). Self-care capacity partially mediated the association between self-perceived burden and QoL (indirect effect: *β* = −0.165, 95% bootstrap *CI*: −0.183 to −0.147, *P* < 0.001). The final model demonstrated good fit(*χ^2^/df* = 2.742, CFI = 0.969, RMSEA = 0.075).

**Conclusion:**

Self-care capacity partially mediates the association between self-perceived burden and QoL in patients undergoing mechanical valve replacement. These findings suggest that interventions targeting both burden reduction and self-care enhancement may help improve QoL, though longitudinal and experimental research is needed to establish causal pathways and confirm clinical implications.

## 1 Introduction

Valvular heart disease (VHD) affects approximately 3.8% of the Chinese population and is a major contributor to heart failure and cardiovascular mortality nationwide (1). For younger and middle-aged patients requiring surgical intervention, mechanical valve replacement remains the standard of care due to superior long-term prosthesis durability (2, 3). Yet this durability comes at a substantial cost. Mechanical heart valves, though more durable than bioprostheses, are more thrombogenic and require lifelong anticoagulation. Patients receiving valve replacement therapy exhibited suboptimal adherence to oral anticoagulant medications, with only 35.6% reporting high levels of adherence (4). The estimated annual incidence of mechanical valve thrombosis and postoperative bleeding in China spans from 0.3% to 1.48% and 0.7% to 10.4%, respectively (5). Beyond these clinical risks, the daily demands of managing a chronic condition—dietary restrictions, activity limitations, frequent blood tests, and persistent worry about complications—place significant psychological strain on patients (6). Over time, these cumulative burdens can erode psychological well-being and disrupt daily functioning, ultimately impacting quality of life. However, the specific pathways through which these clinical demands translate into poorer QoL remain incompletely understood.

Self-perceived burden has received growing attention as a key psychological factor in chronic illness. Studies across cancer, renal disease, and cardiac populations have consistently linked higher burden to poorer QoL, increased depressive symptoms, and lower treatment adherence (7–9). For cardiac surgery patients in particular, the prolonged recovery period and temporary dependency on caregivers can intensify these feelings. In mechanical valve recipients, this burden may be further amplified by the permanent nature of the prosthesis and the lifelong nature of self-management requirements. Yet despite these clinical observations, few studies have examined self-perceived burden specifically in the mechanical valve population, and even less is known about how burden exerts its effects on QoL.

One plausible mechanism is through self-care capacity. Rooted in Orem's self-care deficit theory, self-care capacity refers to an individual's ability to engage in health-promoting behaviors and manage their own condition (10). For mechanical valve recipients, adequate self-care—including medication adherence, dietary modifications, symptom monitoring, and regular physical activity—is essential for preventing complications and maintaining health. While prior work has established self-care as a predictor of QoL in cardiac patients, less is known about what psychological factors shape self-care capacity in the first place.

Drawing on self-regulation theory provides a useful framework for understanding these relationships (11, 12). According to this perspective, self-perceived burden functions as a threat appraisal: when patients perceive themselves as a burden to others, it triggers negative emotions (guilt, shame, anxiety) that consume limited self-regulatory resources. With fewer resources available, patients may struggle to maintain consistent self-care behaviors—a domain that requires ongoing effort, planning, and self-discipline. In turn, reduced self-care engagement leads to worsening symptoms and lower QoL. This mediating pathway—burden→depleted self-regulatory resources→reduced self-care→poorer QoL—has received preliminary support in other chronic illness groups but has not, to our knowledge, been systematically tested in patients following mechanical valve replacement.

This study used structural equation modeling (SEM) to test the hypothesized mediating role of self-care capacity in the relationship between self-perceived burden and QoL among Chinese patients with mechanical valve replacement. By examining both direct and indirect pathways, we aimed to clarify the mechanisms through which self-perceived burden impacts QoL in this population. Such findings could help cardiac nurses design more targeted interventions that address not only the psychological experience of burden but also the behavioral pathways through which it affects health outcomes.

## 2 Methods

### 2.1 Participants

Patients who underwent first-time valve replacement at a tertiary hospital in Guangzhou between December 2022 and December 2023 were recruited using convenience sampling. Patients were approached during their postoperative outpatient follow-up visit, typically 2–4 weeks after hospital discharge. This time point was selected because patients would have had sufficient time to experience the initial challenges of self-management at home while still being in the early recovery phase.

Inclusion criteria were: age≥18 years, absence of cognitive impairment, and adequate communication skills. Exclusion criteria included: (i) Concurrent surgical procedures (e.g., coronary artery bypass grafting, major vascular surgery, or pericardial surgery); (ii) Pre-existing conditions affecting quality of life (e.g., stroke, chronic renal failure, chronic liver failure, or cancer); (iii) Postoperative cardiac complications (e.g., pericardial effusion, paravalvular leakage, severe arrhythmias) or NYHA Class III–IV heart failure/uncontrolled atrial fibrillation.

Sample size was determined based on established recommendations for structural equation modeling. For models with 2∼3 latent variables and moderate effect sizes, a minimum sample size of 200∼400 is generally recommended to achieve sufficient statistical power (≥0.80) for detecting significant paths. Of the 512 eligible patients approached during the study period, 488 agreed to participate (response rate: 95.3%). 26 questionnaires were subsequently excluded due to incomplete responses (>10% missing data) or internal inconsistencies (e.g., contradictory responses on validated attention check items). A final sample of 464 participants was included in the analysis. Ethical approval was obtained from the Institutional Review Board of Guangdong Provincial People's Hospital (Approval number: KY2024-508-03). Written informed consent was provided by all participants or their legal representatives.

### 2.2 Measures

General Information Questionnaire: A researcher-designed questionnaire collected socio-demographic and clinical data, including gender, age, place of residence, marital status, economic status, educational level, payment method for medical expenses, primary caregiver, and surgical approach.

Exercise of Self-Care Agency Scale (ESCAS): Based on Orem's self-care theory, the 43-item ESCAS (13) evaluates four dimensions of self-care capacity: (a) active vs. passive situational responses, (b) motivation, (c) knowledge base, and (d) self-worth perception. We used the validated Chinese version by Wang et al. (14). (Cronbach's *α*= 0.87; content validity index = 1.00). In the present study, Cronbach's *α* was 0.89 for the total scale.

36-Item Short Form Survey (SF-36): The SF-36 (15), developed by RAND in the Medical Outcomes Study (MOS), is a generic patient-reported quality-of-life instrument measuring eight dimensions: physical functioning (PF), role-physical (RP), bodily pain (BP), general health (GH), vitality (VT), social functioning (SF), role-emotional (RE), and mental health (MH). We administered the standardized Chinese translation (16), which demonstrates robust psychometric properties. In our sample, Cronbach's *α* for the overall SF-36 was 0.92.

The Self-Perceived Burden Scale (SPBS): The SPBS developed by Cousineau et al. (17) measures the extent to which individuals perceive themselves as a burden to others across three dimensions: physical burden, economic burden, and emotional burden. The scale consists of 10 items rated on a 5-point Likert scale, with higher scores indicating greater perceived burden. The Chinese adaptation by Zhang et al. (18) was employed, showing high internal consistency (Cronbach's *α* = 0.91). Cronbach's *α* in this study was 0.90.

### 2.3 Data analysis

The first factor accounted for 42.3% of the total variance, which is below the commonly cited threshold of 50%, suggesting that severe common method bias is unlikely in this study. However, some degree of common method variance may still be present and is acknowledged as a limitation.

Data analysis was performed using IBM SPSS Statistics for Windows, version 27.0 (IBM Corp., Armonk, N.Y., USA) and IBM SPSS Amos for Windows, version 26 (IBM Corp.). Continuous variables are presented as mean ± standard deviation. Normality distribution was assessed using Shapiro–Wilk tests. Independent samples *t*-tests and Mann–Whitney *U* tests were applied for normally and non-normally distributed data, respectively.

Pearson correlation coefficients examined bivariate associations among self-perceived burden, self-care capacity, and quality of life. Multiple linear regression was conducted to identify independent determinants of quality of life, with demographic characteristics, clinical variables, self-perceived burden, and self-care capacity entered as candidate predictors.

Structural equation modeling (SEM) was used as the primary analysis to formally test the mediation hypothesis, as it accounts for measurement error and provides a more robust evaluation of direct and indirect pathways compared to regression-based methods. The model was specified with self-perceived burden as an observed exogenous variable (total SPBS score), self-care capacity as a latent endogenous variable (indicated by its four subscales), and quality of life as a latent endogenous variable (indicated by eight SF-36 dimensions). Maximum likelihood estimation was used.

Prior to evaluating the structural model, confirmatory factor analysis (CFA) was conducted to assess the measurement model for each latent variable, ensuring that indicators adequately represented their respective constructs. Model fit was evaluated using multiple indices: *χ*^2^/df ratio (< 3 indicates acceptable fit), comparative fit index (CFI > 0.90), Tucker–Lewis index (TLI > 0.90), root mean square error of approximation (RMSEA < 0.08), and standardized root mean square residual (SRMR < 0.08). Modification indices were consulted to improve model fit where theoretically justified.

Bias-corrected bootstrapping with 5,000 resamples was used to test the significance of the indirect effect. This method is preferred over the Sobel test because it does not assume normality of the sampling distribution and has greater statistical power. An indirect effect was considered statistically significant if its 95% bias-corrected confidence interval did not include zero. The proportion of the total effect mediated was calculated as (indirect effect/ total effect)  ×   100%. Statistical significance was defined as two-tailed *P* < 0.05.

## 3 Results

### 3.1 Participant characteristics

The study included 464 patients who underwent mechanical valve replacement, all with uncomplicated postoperative recovery (no severe complications such as infection, thromboembolism, or hemorrhage). Mean age was 47.29 ± 11.06 years, and 60.5% were male. Most participants were married (79.3%) and reported an average monthly household income below 3,000 RMB (53.9%). Approximately one-third had completed primary school or less (32.1%), while 14.4% held a bachelor's degree or higher. Slightly more than half (59.7%) had a previous medical history of cardiovascular disease. The primary caregiver was most commonly a spouse (56.7%), followed by children (26.3%). Additional demographic and clinical characteristics are presented in detail in Table 1.

**Table 1:** Self-care capability, self-perceived burden, and quality of life scores after valve replacement.

Groups	*n(%)*	ESCAS score	*t/F*	*P*	SF-36 score	*t/F*	*P*	SPBS score	*t/F*	*P*
Gender										
male	281 (60.56)	112.42 ± 18.12	−2.686	0.007	55.44 ± 13.22	7.161	<0.001	32.37 ± 7.09	−1.967	0.05
female	183 (39.44)	117.42 ± 21.69			45.37 ± 13.50			33.76 ± 7.93		
Age group(years)										
≤30	33 (7.11)	124.20 ± 25.67	3.631	0.006	52.45 ± 13.51	6.316	<0.001	30.03 ± 6.99	3.624	0.006
31∼40	108 (23.28)	116.46 ± 20.28			55.64 ± 14.29			33.49 ± 6.88		
41∼50	119 (25.65)	113.06 ± 19.42			51.53 ± 13.61			31.52 ± 7.66		
51∼60	158 (34.05)	113.65 ± 16.92			47.32 ± 13.68			34.16 ± 7.17		
≥61	46 (9.91)	108.51 ± 21.22			48.93 ± 12.95			32.98 ± 8.64		
Education										
Primary school and below	149 (32.11)	113.43 ± 20.04	2.682	0.046	44.96 ± 11.47	34.548	<0.001	33.87 ± 7.01	1.582	0.193
Junior high school	146 (31.47)	112.14 ± 18.84			49.80 ± 13.09			32.57 ± 7.58		
High school	102 (21.98)	115.32 ± 18.80			52.56 ± 14.54			32.86 ± 7.88		
Bachelor's degree or above	67 (14.44)	120.03 ± 21.56			63.74 ± 11.53			31.64 ± 7.41		
Career										
Worker	103 (22.20)	111.93 ± 21.41	4.957	0.002	57.28 ± 12.93	15.526	<0.001	33.60 ± 7.20	2.998	0.030
Famer	95 (20.47)	109.41 ± 19.58			47.89 ± 12.26			33.29 ± 7.81		
Specialist	91 (19.61)	114.91 ± 16.33			53.91 ± 13.64			34.20 ± 7.27		
Others	175 (37.72)	118.28 ± 19.75			47.11 ± 14.16			31.65 ± 7.39		
Marital status										
Married	368 (79.31)	113.25 ± 19.14	−2.446	0.015	51.02 ± 14.29	0.452	0.651	32.96 ± 7.46	0.263	0.793
Others	96 (20.69)	118.76 ± 21.41			50.29 ± 13.08			32.74 ± 7.48		
Residence										
City	307 (66.16)	115.79 ± 19.20	2.141	0.033	51.69 ± 13.67	1.774	0.077	32.60 ± 7.81	−1.339	0.182
Countryside	157 (33.84)	111.66 ± 20.53			49.25 ± 14.63			33.54 ± 6.71		
Payment method										
Medical Insurance	299 (64.44)	114.21 ± 20.28	0.322	0.725	52.98 ± 13.27	13.775	<0.001	32.35 ± 7.43	2.682	0.069
New-Type Rural Cooperative Medical System	131 (28.23)	114.14 ± 19.35			48.51 ± 14.88			33.73 ± 7.52		
Others	34 (7.33)	117.01 ± 16.35			41.34 ± 12.06			32.92 ± 7.46		
Average monthly per capita income of the family(Yuan)										
＜1,000	69 (14.87)	114.72 ± 13.31	5.151	0.002	40.23 ± 11.10	38.684	0.001	32.23 ± 6.88	0.812	0.488
1,000∼2,999	184 (39.65)	111.91 ± 20.66			47.69 ± 13.42			33.55 ± 7.28		
3,000∼4,999	121 (26.08)	120.12 ± 19.34			58.43 ± 11.17			32.43 ± 7.66		
≥5,000	90 (19.40)	111.51 ± 21.05			55.34 ± 13.63			32.81 ± 7.97		
Work condition										
On duty	223 (48.06)	116.82 ± 20.37	4.172	0.016	56.59 ± 13.09	50.678	<0.001	32.22 ± 7.13	2.659	0.071
Retired	89 (19.18)	114.35 ± 15.31			49.67 ± 11.84			34.34 ± 7.63		
Others	152 (32.76)	110.86 ± 20.65			43.17 ± 12.72			33.11 ± 7.45		
Hobby										
Yes	257 (55.39)	116.27 ± 18.75	2.295	0.022	50.48 ± 14.21	−0.657	0.512	31.65 ± 7.27	−4.166	<0.001
No	207 (44.61)	112.06 ± 20.71			51.34 ± 13.83			34.50 ± 7.40		
Previous medical history										
Yes	277 (59.70)	117.01 ± 20.12	3.514	<0.001	51.92 ± 13.61	1.981	0.048	32.00 ± 7.09	−3.261	0.001
No	187 (40.30)	110.52 ± 18.54			49.30 ± 14.54			34.28 ± 7.79		
Primary caregiver										
Children	122 (26.29)	108.93 ± 19.06	14.910	<0.001	47.47 ± 13.72	5.675	0.004	33.73 ± 7.46	1.063	0.346
Spouse	263 (56.68)	114.04 ± 18.97			52.58 ± 13.96			32.72 ± 7.34		
Others	79 (17.03)	123.99 ± 19.99			50.86 ± 14.04			32.33 ± 7.83		

### 3.2 Main outcome scores

Participants reported a mean self-care capacity score of 114.39 ± 19.74 on the ESCAS (possible range: 43∼215, with higher scores indicating better self-care), placing them in the moderate range relative to scale midpoints. Mean self-perceived burden was 32.92 ± 7.46 on the SPBS (possible range: 10∼50, with higher scores indicating greater burden), also falling in the moderate range. Quality of life scores on the SF-36 averaged 50.86 ± 14.04 (possible range: 0∼100, with higher scores indicating better QoL), suggesting moderate overall quality of life in this patient group.

### 3.3 Bivariate correlations

Pearson correlation analysis demonstrated significant negative associations between self-perceived burden and both self-care capacity (*r* = −0.760, *P* < 0.001) and quality of life (*r* = −0.826, *P* < 0.001). Conversely, self-care capacity positively correlated with quality of life (*r* = 0.822, *P* < 0.001) (Table 2). These bivariate associations provided initial support for the hypothesized mediation model.

**Table 2:** Correlation analysis of patients' self-care capacity, self-perceived burden, and quality of life in patients undergoing valve replacement.

	self-care capacity	self-perceived burden	Quality of Life
self-care capacity	1		
self-perceived burden	−0.760**	1	
Quality of Life	0.822**	−0.826**	1

** *P < *0.001.

### 3.4 Multiple regression analysis

Stepwise multiple regression identified five independent predictors of quality of life: age, monthly household income, previous medical history, self-perceived burden, and self-care capacity (Table 3). The full model accounted for 85.9% of the variance in QoL (*R^2^* = 0.859, adjusted *R^2^* = 0.849, *F* = 127.243, *P* < 0.001). Higher income was the strongest positive predictor, while older age and previous medical history were associated with poorer QoL (all *P* < 0.05). Greater self-perceived burden independently predicted poorer QoL (*β* = −0.241, *P* < 0.001), whereas better self-care capacity predicted better QoL (*β* = 0.137, *P* < 0.001). Multicollinearity diagnostics indicated no significant issues, with all variance inflation factor (VIF) values below 3 (range: 1.12∼2.87) and all tolerance values above 0.34.

**Table 3:** Results of multivariate regression analysis of influencing factors on the quality of life of patients undergoing mechanical valve replacement.

Independent variable	*B*	*S.E.*	*β*	95%CI for *B*	*t*	*P*
Constant	56.375	7.442	-	[41.79, 70.96]	9.346	<0.001
Age group (reference: ≤30 years)						
31∼40 years	3.124	1.356	0.103	[0.47, 5.78]	3.316	<0.001
41∼50 years	−5.633	1.251	−0.121	[−8.09, −3.18]	−4.162	<0.001
51∼60 years	−10.480	2.146	−0.173	[−14.69, −6.27]	−5.327	<0.001
≥61 years	−6.180	5.188	−0.108	[−16.36, 3.99]	−3.244	0.002
Income group (reference: <1,000 Yuan/month)						
1,000∼2,999Yuan/month	8.503	1.247	0.220	[6.06, 10.95]	6.322	<0.001
3,000∼4,999Yuan/month	13.338	1.982	0.385	[9.45, 17.22]	9.160	<0.001
≥5,000Yuan/month	19.672	2.667	0.401	[14.45, 24.90]	10.019	<0.001
Previous medical history(Yes)	−5.004	2.364	−0.105	[−9.64, −0.37]	−2.240	0.005
self-care capacity	0.323	0.104	0.137	[0.12, 0.53]	3.636	<0.001
self-perceived burden	−0.541	0.083	−0.241	[−0.70, −0.38]	−6.515	<0.001

*R^2^* = 0.859, *ΔR^2^* = 0.849, *F* = 127.243, *P < *0.001.

Given their independent effects, structural equation modeling was performed to further examine the potential mediating pathway between these psychosocial factors and QoL.

### 3.5 Mediating effect of self-care capacity

**Figure 1:**
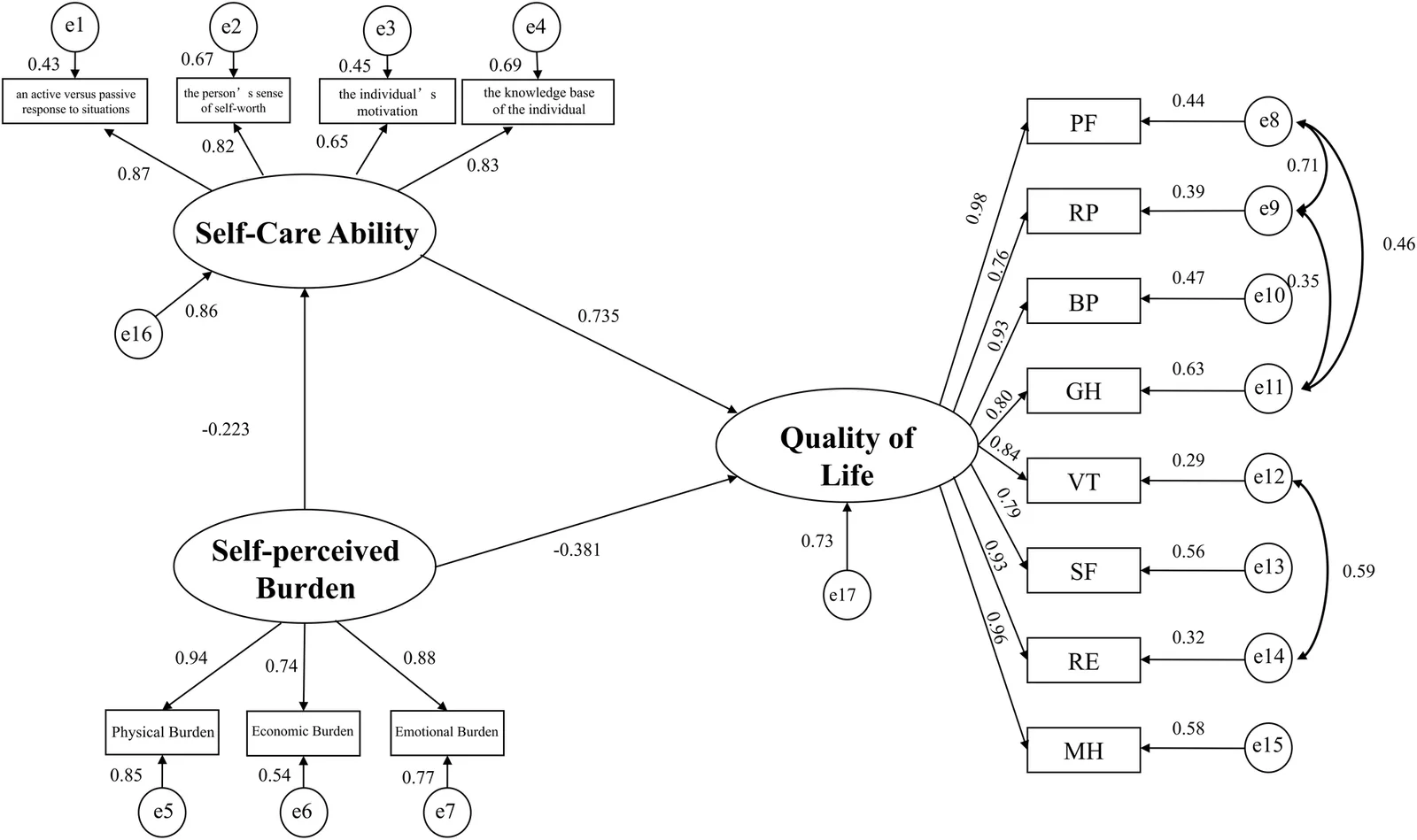
Structural equation model illustrating the mediating role of self-care capacity in the relationship between self-perceived burden and quality of life among patients following mechanical valve replacement (N = 464). Rectangles represent observed variables; ovals represent latent variables; small circles with numbers represent error terms. Numbers on single-headed arrows indicate standardized path coefficients. e1-e4: subscales of the Exercise of Self-Care Agency Scale (active versus passive situational response, knowledge base, the person's sense of self-worth, and motivation); PF: physical functioning; RP: role-physical; BP: bodily pain; GH: general health; VT: vitality; SF: social functioning; RE: role-emotional; MH: mental health.

Structural equation modeling was conducted to test the mediating role of self-care capacity in the association between self-perceived burden and quality of life. The final model showed good fit: *χ*^2^/df = 2.742 (<3), RMSEA = 0.075 (<0.08), CFI = 0.969, TLI = 0.910, SRMR = 0.059, with all key indices meeting acceptable thresholds (Figure 1). As shown in the model, self-perceived burden had a significant negative predictive effect on self-care capacity (*β*= −0.223, *P* < 0.001), and self-care capacity had a significant positive predictive effect on quality of life (*β*= 0.735, *P* < 0.001). After controlling for self-care capacity, self-perceived burden still exerted a significant direct negative effect on quality of life (*β*= −0.381, *P* < 0.001).

The results of Bootstrap analysis are presented in Table 4. The indirect effect of self-perceived burden on quality of life through self-care capacity was −0.164 (95% *CI*: −0.269 to −0.063, *P* < 0.001), accounting for 30.1% of the total effect. Since the 95% confidence interval did not contain zero, self-care capacity played a partial mediating role between self-perceived burden and quality of life.

**Table 4:** Mediating effect of self-care capacity on the relationship between self-perceived burden and quality of life.

Type of effect	Path	Standardized *β*	95% *CI*	*P*
Direct effects	SPB → QoL	−0.381	[−0.503, −0.259]	<0.001
Indirect effect	SPB → Self-care capacity → QoL	−0.164	[−0.269, −0.063]	<0.001
Total effect	SPB → QoL	−0.545	[−0.687, −0.403]	<0.001
—	Proportion mediated (%)	30.1%	—	—

## 4 Discussions

Patients undergoing mechanical valve replacement face unique long-term challenges, and the mechanisms linking self-perceived burden to their quality of life remain incompletely understood. This study examined whether self-care capacity mediates this relationship.

### 4.1 The direct relationship between SPB and QoL

The finding that SPB is directly and negatively associated with QoL aligns with previous research across various chronic illness populations (7, 8, 19). Patients often rely on family members for daily care during the initial postoperative months, so they are easy to feel like a burden to their family.

According to the conceptual framework of perceived burden by Cousineau et al. (17), the experience of burden encompasses emotional dimensions such as guilt, shame, and diminished self-worth, which can directly erode QoL. Additionally, patients with high SPB may withdraw from social interactions to avoid burdening others, leading to social isolation and further worsening of QoL (20). Our future research could further include measures of psychological distress and social support to better understand the pathways underlying this effect.

### 4.2 The mediating role of self-care capacity

This study found that self-care capacity partially mediates the relationship between SPB and QoL, explaining roughly 30% of the total effect. This finding helps clarify how SPB impacts QoL in valve replacement patients.

Why does self-care capacity play a partial mediating role? We think two possible explanations may help interpret this finding. First, the Common-Sense Model of Self-Regulation (CSM) suggests that patients' illness perceptions shape their self-management behaviors and subsequent outcomes (12). When patients feel like a burden, they may underestimate their ability to manage their health, leading to reduced engagement in self-care activities. Second, from a social cognitive perspective, SPB may diminish their self-efficacy—the confidence in one's ability to perform self-care tasks—thereby decreasing participation in rehabilitation, symptom monitoring, and medication management (21). This matches what He et al. (7) found in coronary heart disease patients: self-efficacy mediated the link between SPB and kinesiophobia. For mechanical valve replacement patients, who have complex postoperative self-care needs, interventions targeting both perceived burden and self-care ability may be more effective than addressing either factor alone.

Our finding that better self-care is linked to improved QoL is supported by intervention research showing that programs combining communication support with rehabilitation can enhance both self-care ability and quality of life in valve surgery patients (22). The relatively strong association between self-care capacity and QoL observed in this study likely reflects the central role of self-management in postoperative recovery. However, the possibility of common method variance should be considered, as both variables were measured by self-report.

### 4.3 Implications and future research

These findings have some practical implications. First, routine assessment of SPB could help identify mechanical valve replacement patients at higher risk for poor QoL, though further research is needed to confirm whether screening and targeted interventions improve outcomes. Second, since self-care capacity partially mediates the effect of SPB on QoL, it represents a modifiable target for intervention. Previous studies have shown that empowerment-based self-care education works in heart failure patients (23), and similar approaches may be worth exploring in valve replacement populations. A meta-analysis of transitional care for valve surgery patients also found that programs supporting self-management improved both anticoagulation control and QoL outcomes (24).

Two key areas warrant further investigation. First, intervention studies testing whether programs targeting both SPB and self-care capacity can improve QoL would be particularly valuable, as this would provide direct evidence for clinical practice. Mobile-based interventions, for example, have shown feasibility in improving self-care among valve replacement patients in recent randomized trials (25). Second, research exploring how to best implement self-care support in routine clinical care for valve replacement patients is also needed.

### 4.4 Limitations

Several limitations should be acknowledged.

First, the cross-sectional design prevents definitive conclusions about causal relationships. Although we hypothesized that SPB leads to reduced self-care capacity and poorer QoL, reverse or reciprocal relationships are also plausible. Longitudinal or interventional studies are needed to confirm the direction of effects.

Second, all measures were self-reported, which may introduce common method bias. The strength of the self-care capacity-QoL association, in particular, may be partially inflated by shared method variance. Future studies could incorporate objective measures of self-care or clinician-rated outcomes to complement self-report data.

Third, this study was conducted at a single tertiary hospital, which may limit generalizability to other healthcare settings or patient populations. Multi-center studies with more diverse samples would strengthen external validity.

Fourth, this study is subject to common method variance, as all data were collected through self-report questionnaires at a single time point. Although Harman's single-factor test suggested no severe common method bias (first facto*r* = 42.3%), we cannot completely rule out this potential influence. Future studies using multi-source or longitudinal designs would help address this limitation.

Finally, while the mediation model identifies self-care capacity as a mechanism, the specific psychological processes through which SPB affects self-care (e.g., reduced self-efficacy, illness perception biases) were not directly measured. Future research examining these intermediate variables would help clarify the specific pathway, and exploring additional mediators beyond self-care should also be investigated to fully understand the SPB-QoL relationship.

### 4.5 Conclusions

In conclusion, our study shows that self-care capacity plays an important role in how self-perceived burden affects quality of life in mechanical valve replacement patients. This finding suggests that paying attention to both patients' psychological burden and their self-care ability may help improve their postoperative quality of life. More research is needed to develop and test effective interventions for this population.

## Data Availability

The raw data supporting the conclusions of this article will be made available by the authors, without undue reservation.
